# The Role of Essential Oils in Sports Recovery and Performance

**DOI:** 10.3390/molecules30183771

**Published:** 2025-09-17

**Authors:** Stanislava Ivanova, Zoya Dzhakova, Yana Gvozdeva, Gergana Petkova, Albena Ivanova, Elizabet Dzhambazova

**Affiliations:** 1 Department of Pharmacognosy and Pharmaceutical Chemistry, Faculty of Pharmacy, Medical University of Plovdiv, 4002 Plovdiv, Bulgaria; zoya.dzhakova@mu-plovdiv.bg; 2 Research Institute, Medical University of Plovdiv, 4002 Plovdiv, Bulgaria; yana.gvozdeva@mu-plovdiv.bg; 3 Department of Pharmaceutical Technology and Biopharmacy, Faculty of Pharmacy, Medical University of Plovdiv, 4002 Plovdiv, Bulgaria; 4 Department of Languages and Specialized Training (DLST), Medical University of Plovdiv, 4002 Plovdiv, Bulgaria; gergana.petkova@mu-plovdiv.bg (G.P.); albena.ivanova@mu-plovdiv.bg (A.I.); 5 Department of Medical Informatics, Biostatistics and E-Learning, Faculty of Public Health, Medical University of Plovdiv, 4002 Plovdiv, Bulgaria; elizabet.dzhambazova@mu-plovdiv.bg

**Keywords:** essential oils, lavender essential oil, peppermint essential oil, sports medicine, traditional medicine

## Abstract

Recent clinical studies and scientific literature increasingly support the use of essential oils (EOs) as adjuncts in enhancing sports performance and recovery. They have demonstrated potential in modulating mood, alleviating fatigue, facilitating muscle recovery, and contributing to the overall physiological and psychological well-being of athletes. Specifically, EOs such as peppermint and eucalyptus exhibit analgesic and anti-inflammatory properties, making them beneficial for managing exercise-induced muscle soreness and discomfort. Conversely, oils like lavender are recognized for their anxiolytic and sedative effects, which may improve sleep quality and promote relaxation. But both are essential for effective post-exercise recovery. As such, their strategic application may represent a valuable, complementary approach within the broader context of sports medicine and athletic training. Although EOs have been used for centuries in traditional medicine of various cultures to support physical performance, scientific research in the field of sports medicine remains still limited. Preliminary findings suggest promising effects on fatigue reduction, sleep, sympathetic activity, and endurance improvement. However, results are inconsistent, with some studies even showing no significant differences compared to placebo. Further rigorous research is needed to establish the efficacy and mechanisms of EOs in athletic settings.

## 1. Introduction

Aromatic plants have played a fundamental role in traditional medicine and as well as in the daily life of human beings for centuries [1,2,3,4,5,6]. Today, these plants are increasingly recognized as valuable sources of bioactive compounds with significant therapeutic potential, which may be used for improving human well-being and for advancing medical progress [7,8]. Moreover, it is also anticipated that several innovative pharmaceutical agents may be derived from aromatic plants in the coming/forthcoming decade. Due to the necessity for creative approaches to fully harness the therapeutic benefits of the natural substances mentioned, current scientific interest in the study of aromatic plants is substantial and growing [7]. Essential oils (EOs), which represent complex natural mixtures composed of volatile organic compounds, are predominantly synthesized by aromatic plants as secondary metabolites [4]. These compounds belong to different chemical classes: alcohols, phenols, aldehydes, ketones, ethers or oxides, esters, amides, amines, heterocycles, and primarily terpenes, including monoterpene hydrocarbons and oxygenated monoterpens, as well as sesquiterpene hydrocarbons and oxygenated sesquiterpens [9]. In most cases, EOs contain one or a few dominant components, with additional compounds present in smaller amounts. The chemical composition may vary, influenced by various factors, such as plant parameters, plant origin, soil conditions, climate, harvest period, and others/etc. [10]. These compounds serve crucial ecological roles, such as repelling herbivores and attracting pollinators. EOs are characterized by their distinctive scents and exhibit a broad spectrum of biological activities, including antibacterial, antifungal, antiviral, anticancer, anti-inflammatory, antioxidant, and insecticidal properties [9,11]. That is the reason why they can also be used in the context of sports and athletic performance, in order to enhance both physical and mental health. While they are not a substitute for proper training, nutrition, and recovery practices, EOs may complement an athlete’s routine in different ways.

It is considered that the term “essential oils” has been utilized since the 16th century, originating from the words “Quinta essentia”, which is literally translated from Latin to “fifth essence”, “fifth element”, or “aether”. The phrase consists of two lexemes: the feminine gender form of the numeral (or more precisely, ordinal number) quintus, -a, -um, which means “fifth”; and the first declension noun essentia, -ae, f—or “essence”—added [12]. The therapeutic practice that promotes physical and mental well-being using EOs is known as aromatherapy.

The aim of the present research is to introduce and assess the role of EOs in enhancing sports recovery and performance. The manuscript addresses a significant gap by identifying and analyzing the limited number of clinical studies that directly investigate the relationship between EOs and sports medicine. Furthermore, it synthesizes indirect evidence from related research to support the potential roles of EOs in areas such as performance enhancement, recovery, pain management, and psychological well-being in athletes. By consolidating both direct and indirect evidence, the manuscript provides a comprehensive and novel perspective on the therapeutic potential of EOs in modern sports medicine. It also highlights the potential usage of key aromatic plants relevant to sports medicine and outlines various methods of EOs application.

## 2. Results and Discussion

### 2.1. Key Aromatic Plants in Sports Medicine: Therapeutic Uses and Benefits

Aromatic plants are increasingly recognized in sports medicine for their therapeutic effects that support athletic performance, recovery, and injury prevention [13,14]. EOs derived from these plants offer various benefits, such as alleviating pain, promoting muscle relaxation and recovery, enhancing circulation and physical performance, improving mental focus, reducing stress, and aiding skin regeneration. Key aromatic plants commonly used in this context include *Arnica montana* L., *Matricaria chamomilla* L., *Eucalyptus globulus*, *Lavandula angustifolia* Mill., among others (Table 1).

*Arnica montana* L., commonly known as Arnica, is a plant whose name means “lamb’s skin” due to the texture of its leaves [39]. It naturally grows in grasslands, shrublands, and alpine regions, but can also be found in dry pine woodlands, meadows with siliceous soils, the edges of spruce forests, open woodland margins, hay pastures, and the borders of peatlands [40]. The plant contains several bioactive compounds, including sesquiterpene lactones, terpenoids, flavonoids, phenolic acids, and EOs, which are known for their antioxidant, anti-inflammatory, antibacterial, antifungal, antiseptic, antiradical, and antisclerotic properties. The composition of its EOs, particularly the major volatile constituents, can vary significantly depending on geographic location and environmental conditions. EOs extracted from various plant parts of Arnica specimens collected across Europe predominantly contain sesquiterpene hydrocarbons (such as *E*-caryophyllene, germacrene D, α-humulene, and bicyclogermacrene), oxygenated monoterpenoids (e.g., 1,8-cineole, linalool), oxygenated sesquiterpenoids (e.g., caryophyllene oxide, α-cadinol), and phenyl derivatives (such as 2,5-dimethoxy-*p*-cymene, thymol methyl ether, *p*-methoxyheptanophenone, and 2,6-diisopropylanisole). According to the literature data, the chemical profile of EO can differ depending on whether it is extracted from flower heads, rhizomes, roots, or root hairs, suggesting a wide range of potential applications due to the diversity of secondary metabolites present [15].

Thymol, a phenolic monoterpenoid, is widely used in the pharmaceutical and food industries. Thymol and its derivatives exhibit a wide range of biological activities, such as anti-inflammatory, analgesic, antibacterial, antifungal, antiviral, antipyretic, anticancer, anticonvulsant, antitumor, and antidepressant effects [41]. The anti-inflammatory activity of the compound is associated with reduced levels of Tumor Necrosis Factor-α (TNF-α) and interleukins. TNF-α contributes to the processes of vasodilation, edema formation, and promotion of the process of leukocyte adhesion to the epithelial surface by activating adhesion molecules. In addition, TNF-α is involved in blood coagulation, induces oxidative stress at sites of inflammation and indirectly leads to fever. Interleukins also serve as mediators of the inflammatory response in vascular and immune cell lines, playing an important role in the progression of vascular inflammatory conditions. The main role of interleukins is to regulate cell activation, growth, and differentiation during immune and inflammatory responses [42]. Thymol also possesses analgesic activity related to its local anesthetic properties. In vitro, a blocking effect on voltage-activated sodium channels has been established in various experimental cell model systems from animals and humans (skeletal muscle and neuronal sodium channels) [43]. Moreover, this component is thought to act at different phases of the wound healing process by stimulating re-epithelialization, angiogenesis, and granulation tissue, improving collagen deposition and modulating the growth of fibroblasts and keratinocytes [42]. Thymol was found to be the most effective antibacterial compound in comparison with carvacrol, trans-cinnamic acid, eugenol, and diacetyl against *Escherichia coli* and *Salmonella enterica sero* var. *typhimurium* [43]. Research indicated that the antibacterial properties were related to the ability to induce permeabilization and depolarization of the cytoplasmic membrane [43].

*Matricaria chamomilla* L. (Asteraceae) is a popular and well-known medicinal plant. Nowadays, the herb is widely used in traditional medicine, as well as in the cosmetic and food industries [44]. The word chamomille (*Matricaria chamomilla* L.) is associated with the plant’s apple-like aroma (meaning: apple-of-the-ground), as it was described for the first time by Dioscorides [39]. The Latin word “matrix” (womb) gives the name of the genus *Matricaria*, because of its usage in ancient herbal medicine for the treatment of cramps and sleep issues associated with premenstrual syndrome [39,44]. The plant is used for its known pharmacological activities, such as anti-inflammatory, antispasmodic, carminative, sedative, antiseptic, antifungal, as well as antibacterial activity against Gram-positive and Gram-negative microorganisms [44,45,46]. Topical formulations containing chamomile show considerable effectiveness in the treatment of different skin conditions, along with the acceleration of the wound healing process [19]. Precisely because of the fact that restoring tissue integrity is a dynamic and complex process, chamomile with its multitarget activities is quite suitable for such an application [19]. The EO of the plant’s inflorescence is also used as an analgesic and muscle pain relief substance [20]. In addition, a decoction of the whole plant or stem is applied topically for sprains and broken bones [20].

*Cinnamon* is typically derived from the bark of tropical trees belonging to the genus Cinnamomum, while cinnamon EO is extracted through hydrodistillation of the bark [47]. It has been known since ancient times and is used to these days for a broad range of medical conditions [48]. The etymology of *Cinnamomum verum*, commonly known in breve as cinnamon, is connected with the Greek word “κινναμωμον” (meaning spice) in combination with the Latin lexeme “*verum*”, a neutral gender form of the adjective verus, -a, -um, which means “true” [39]. The cinnamon EO is associated with strong antioxidant, anti-inflammatory, antimicrobial, antifungal, and antispasmodic activity [48,49]. Cinnamaldehyde isolated from cinnamon EO composition is associated with rapid muscle recovery. Thus, it is used as an alternative supplement for athletes to improve their performance, as it reduces muscle soreness and pain, which are related to inflammation [21,24]. Eugenol, a well-documented analgesic acting compound in cinnamon EO, is known to alleviate muscle pain by blocking ion channels associated with pain transmission. It was found to inhibit high-voltage-activated calcium channels (HVACCs), which contributed to its pain relief effect [50]. Furthermore, eugenol has been identified as an inhibitor of the transient receptor potential vanilloid 1 (TRPV1) receptor, leading to increased levels of intracellular Ca^2+^, which lead to a reduction in pain sensations. Additionally, the compound blocks the activity of lipoxygenase and cyclooxygenase, reducing inflammation and oxidative stress, which are associated with muscle pain [50]. Cinnamon EO decreases muscle damage by improving changes in creatine kinase (CK) levels [51]. Oxidative damage caused by intense exercises could be minimized with prolonged supplementation with cinnamon [22].

*Eucalyptus globulus* is commonly introduced as eucalyptus or gum tree. The phytonym coinage is associated with the fact that the calyx operculum conceals the flower parts at first. The name of the species is derived from the Latin word “*globulus*”, meaning “small ball”/”sphere”, that describes the shape of its fruit [39]. As a member of the Myrtaceae family, this plant produces one of the most notable EOs. Eucalyptus EOs inhalation has been shown to support respiratory health and to affect bronchial infections, rhinitis, and fever reduction [27]. Eucalyptol, or 1,8-cineol, is a major oxygenated monoterpene found in the chemical composition of eucalyptus EO [27]. The compound has shown notable antioxidant and anti-inflammatory activities, reducing the production of reactive oxygen species (ROS) and inflammatory cytokines [52,53]. 1,8-Cineole is used in the treatment of respiratory conditions due to its anti-inflammatory properties, promotes muscle relaxation, and suppresses excessive mucus production, resulting in decreased levels of inflammatory cytokines like interleukin-1b (IL-1b) and TNF-α [52]. The anti-inflammatory activity of 1,8-cineole is associated with the nuclear transcription factor—NF-jB. As a tracheal muscle relaxant, its effect is most likely associated with the blockade of L-type voltage-gated calcium channels. Supporting the respiratory function, 1,8-cineole has a proven therapeutic effect in rhinosinusitis, asthma, and chronic obstructive pulmonary disease (COPD) [52]. Additionally, the compound displays pain-relieving, anesthetic, sedative, antifungal, and antimicrobial properties against *Staphylococcus aureus*, *Escherichia coli*, and *Pseudomonas aeruginosa* [28,52]. It is also used in aromatherapy to reduce stress and anxiety [28]. Based on its antioxidant and anti-inflammatory properties, topical eucalyptol ointment demonstrates notable wound healing capabilities [29]. Moreover, *Eucalyptus* EO, applied topically or as part of massage oils, relieves joint discomfort in rheumatism, as well as muscle discomfort in muscle strains and sprains [28].

**Figure 1:**
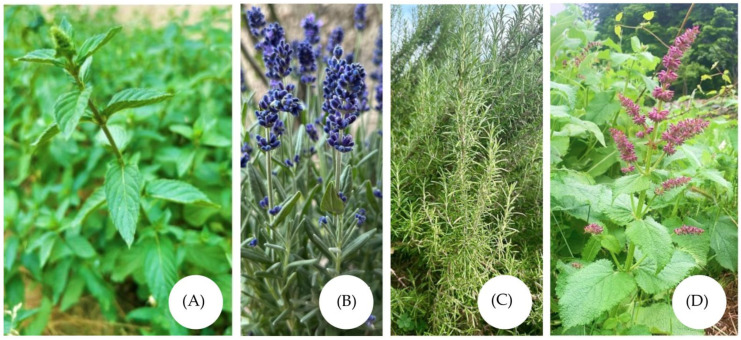
*Mentha piperita* L. (**A**), *Lavandula angustifolia* (**B**), *Salvia rosmarinus* (**C**), *Salvia verticillata* (**D**).

*Mentha piperita* L. (Figure 1A) is a popular aromatic herb, whose name is derived from the Latin word of Greek origin, “*menta/menthe*” (“*mint*”), that comes from the Greek lexeme “minthē” with unclear meaning (probably a loan-word from a lost Mediterranean language) [39]. The phytomym is taken as a personified image for a nymph transformed into a herb by Proserpine and is probably named after her [54]. The Latin word “*piperita*” is a feminine gender form of the adjective piperitus, -a, -um—“hot”. The herb belongs to Lamiaceae family. It is a perennial aromatic plant, associated with antioxidant, anti-inflammatory, analgesic, decongestant, and antispasmodic activity [32]. The aroma of peppermint has a significant impact on stress and anxiety, while also enhancing concentration and attention processes [32]. Menthol, the main component of *Mentha piperita* EO, is responsible for the characteristic fresh aroma and taste, as well as for the cooling effect when applied topically to the skin and mucous membranes. These effects result from stimulation of the membrane-bound ion channel transient receptor potential melastatin 8 (TRPM-8), which reflects changes in temperature in the range of 8–28 °C. During heat stress, stimulation of these receptors improves thermal comfort and reduces the sensation of heat. In addition to improving altered body temperature regulation, menthol can enhance subjective nasal patency, changes in blood flow, and also reduce thirst. For this reason, it is included in a number of products, from oral hygiene products to topical analgesics [55], affecting neuropathic pain as well [34]. Peppermint massage oil relieves muscle pain and soreness and speeds up recovery after exercise [34]. Additionally, inhaling the EO makes lung capacity and breathing better by increasing the force of nasal airflow. It is assumed that this effect leads to a greater delivery of oxygen to the brain, potentially benefiting physical endurance [32]. Furthermore, a study proves that adding the oil to athletes’ water and taking it before and during a competition leads to improved physical performance by increasing the time to exhaustion without changing the level of hydration [33]. The mixture of peppermint and eucalyptus EO improves cognitive abilities, leads to muscle relaxation, and reduces stress [56].

*Lavandula angustifolia* Mill. (Figure 1B) is a flowering plant, known also as lavender, true lavender, garden lavender, common lavender, and narrow-leaved lavender. The genus name, *Lavandula*, is derived from the diminutive of the Latin word “*lautum*” (washed), a past participle from the verb lavo, lavare (to wash), due to its use in the cleansing process. Based on the plant’s slender leaves, it is declared to be “*angustifolia*” (meaning “narrow-leaved”) [39]. Lavender EO holds notable importance in the fields of cosmetics, fragrance, and pharmaceutical industries [31]. It is usually found in various scents and products such as perfumes, soaps, skincare lotions, aromatherapy treatments, and food flavorings [31]. *Lavandula angustifolia* EO possesses different pharmacological activities, such as antioxidant, anti-inflammatory, antimicrobial, antifungal, anxiolytic, anticholinesterase, and anticonvulsant effects [57]. Furthermore, studies have demonstrated that low-dose topical and oral use of the oil produces anti-inflammatory and anti-edema effects, whereas higher doses may cause irritation [57]. Some of the main compounds found in lavender EO are linalool and linalyl acetate. These compounds are essential for the biological activity of this EO. Linalool is a naturally derived monoterpene alcohol, associated with significant anti-inflammatory effects by influencing critical signaling cascades associated with inflammation. Its primary mechanism involves the inhibition of the mitogen-activated protein kinase and nuclear factor kappa-light-chain-enhancer of activated B cell pathways, both of which play central roles in the expression of inflammatory mediators. Furthermore, linalool has been shown to decrease the production of key pro-inflammatory cytokines, such as tumor necrosis factor-alpha and interleukin-6, as well as to suppress nitric oxide synthesis, thereby reducing overall inflammatory activity. The anti-inflammatory mechanism of action of linalyl acetate is similar to linalool. Many scientists consider these compounds as promising natural anti-inflammatory agents [58,59,60]. Inhalation of linalool can extend sleep duration, promote sedation and relaxation, and reduce aggression. Furthermore, both linalool and lavender EO demonstrate calming effects by decreasing activity in renal sympathetic nerves and enhancing parasympathetic nerve activity. The sedative properties of linalool may be influenced by its ability to modulate glutamatergic neurotransmission. The compound acts as a competitive antagonist of the excitatory neurotransmitter glutamate by attaching to glutamatergic N-methyl-D-aspartate (NMDA) receptors [61].

*Zingiber officinale* Rosc., ginger or ginger root, is a word derived from the Greek word ξιϒϒιβερις, itself derived from a Sanskrit name, “singabera” or “shrigavera”, where “srngam” means “horn”, and “vera” means “body” (defining the root’s shape) [39]. Ginger has long been recognized as a medicinal plant, traditionally used in Ayurvedic and Chinese medicine. Today, ginger EO is commercially extracted from both fresh and dried rhizomes. Key bioactive constituents of ginger EO include zingiberene, citral, β-bisabolene, geranial, and camphene. Ginger EO exhibits a wide range of pharmacological effects, including anti-inflammatory, analgesic, anticancer, antioxidant, antinociceptive, and antitussive activities. Advances in cultivation and extraction technologies have also led to the development of ginger cultivars with improved oil yield and composition [30]. Gingerols from the chemical composition of *Zingiber officinale* inhibit the function of COX enzymes and the production of leukotrienes and prostaglandins. In addition, they are agonists of the TRPV1, which is found in the central and peripheral nervous system and thus affects pain. Moreover, ginger inhibits the release of pro-inflammatory cytokines from the macrophages [62]. Due to their influence on COX enzymes, a decrease in muscle pain and soreness is observed, but the effect on muscle function is minimal. It may also lead to a reduction in the elevated production of muscle proteins that typically occurs during exercise. Therefore, prolonged use of ginger results in changed morphological and functional responses to endurance training [62]. Ginger may help alleviate exercise-induced bronchoconstriction, as it acts as a bronchodilator, possibly by affecting the β-adrenergic receptor and altering intracellular Ca^2+^ in airway smooth muscle cells [62]. Citral has also been shown to affect significant inflammatory pathways, such as COX-2 and NF-κB inhibition, reduction of pro-inflammatory cytokines, and activation of peroxisome proliferator-activated receptors (PPARs). This reduces inflammation in a variety of systems, including respiratory, gastrointestinal, neuroinflammatory, and orofacial disorders [63]. It has been associated with improved performance during repeated high-intensity exercises [64].

*Salvia rosmarinus* Spenn. (Figure 1C) is an aromatic plant, whose name “rosemary” originates from the Latin words for “dew” and “sea”. It was introduced for the first time by Pliny and it is supposed to be derived from the Latin *ros maris* (ros, roris, m—“dew; light rain; spray/splash water”, as well as the genitive singular form of mare, maris, n—“sea”, or the masculine gender form of the adjective marinus, -a, um—“belonging to the sea”), for a plant of dewy places (in English, it became Mary’s rose, or rosemary) [39]. The plant is associated with nutritional benefits, a wide range of medicinal properties, and various applications. It is often used in both traditional medicine and the food industry [1,65]. Eucalyptol is one of the main components of the EO and it is associated with strong anti-inflammatory potential and antioxidant activity [28,52,53]. The compound is regarded as a valuable agent in the treatment of different chronic diseases. It was established that eucalyptol inhibits the production of pro-inflammatory cytokines triggered by lipopolysaccharide, including NF-κB, TNF-α, IL-1β, and IL-6, by modulating the extracellular signal-regulated kinase pathway. It also alleviates oxidative stress by regulating signaling pathways and acting as a radical scavenger [66]. The biological activity of the compound was investigated in various cell cultures, animal models, and patients suffering from chronic illnesses. Additionally, due to its ability to cross the blood–brain barrier, eucalyptol can serve as a delivery vehicle for drugs to the brain using microemulsion systems [66]. Camphor, which is another major component of rosemary EO, possesses analgesic, antinociceptive, antimicrobial, antiviral, insecticidal, antitussive, and anticancer activities. Currently, the compound is found in rubefacients and topical analgesics for relieving muscle pain. Camphor is also used for the topical treatment of infections and as a topical antipruritic [1]. With its proven local anesthetic potential, camphor affects skin diseases such as acne, psoriasis, eczema, ulcers and wounds, inflammations and fungal infections, along with effectiveness against swelling [67]. Camphor has been reported to enhance heart function and peripheral circulation. In cases of heart failure and collapse, which present with cold skin, a weak pulse, and heart dysfunction, camphor EO is noted to induce skin reddening, widen peripheral blood vessels, and boost overall circulation [68]. The compound influences various types of receptors—heat-sensitive TRPV1, cold-sensitive Transient receptor potential melastatin 8 (TRP-M8), and heat-sensitive Transient receptor potential vanilloid 3 (TRPV3)—while it also acts as an inhibitor of Transient receptor potential ankyrin 1 (TRPA1). TRP ion channels contain regions that are sensitive to temperature, known as thermoTRP. The interaction of this compound with TRPV1 is associated with a desensitizing effect that leads to pain relief. Research has also been conducted on its effects on blood circulation and the perception of temperature, including both heat and cold [67]. Aromatherapy with rosemary EOs is regarded as a successful approach for alleviating fatigue and decreasing stress hormone levels. Physical therapies, including massage with rosemary EO and acupoint acupressure, were effective in reducing stress and fatigue arising from muscle damage and discomfort [38]. The EO demonstrates more rapid ability to relieve muscle spasms than peppermint EO [37].

The EOof *Salvia verticillata* (Figure 1D) remains relatively unexplored; however, it exhibits promising potential for applications in sports medicine. This EO is particularly abundant in β-caryophyllene (BCP) [69,70], a cannabinoid type-2 receptor agonist known for its wide-ranging therapeutic properties. Nowadays, BCP is regarded as a promising candidate for neuroprotection [71] and is well recognized for its anti-inflammatory activity, achieved through the suppression of key mediators such as inducible nitric oxide synthase, interleukin-1β, interleukin-6, nuclear factor kappa B, and the cyclooxygenase enzymes COX-1 and COX-2. These anti-inflammatory effects are partly mediated by the activation of peroxisome proliferator-activated receptors, particularly PPAR-α and PPAR-γ. Although no studies on which the role of *Salvia verticillata* EO in sports medicine are available at that moment, its composition suggests considerable potential in this research field. This EO contains also rosmarinic acid, γ-muurolene, limonene, α-humulene, germacrene, and others. Both in vivo and in vitro studies on *S. verticillata* extracts and EOs have demonstrated noteworthy antioxidant, anti-inflammatory, antibacterial, and antifungal properties [4].

### 2.2. Integrating Essential Oils into Athletic Training and Physical Performance

The impact of EOs on individuals could be classified into two main categories: physiological and psychological effects. Physiological effects influence the body directly, while psychological effects occur through the olfactory system, where the sense of smell can indirectly trigger physical responses [64].

EOs can be applied through three main pathways: inhalation through the olfactory system, topical application on the skin (a primary route, as the chemical compounds are absorbed through the skin), and oral ingestion, either by drinking or consuming them through the digestive system [72].

#### 2.2.1. Aromatherapy

Although there is growing interest in the effects of EOs on athletic performance observed, evidence of a clear and significant direct impact remains still limited. Physical exercise disrupts the body’s homeostasis, leading to increased muscle metabolism and, consequently, greater demands on the circulatory and respiratory systems. To counteract or delay fatigue, athletes often complement structured training programs with additional strategies, including nutritional supplements and aromatherapy [73].

Aromatherapy is a holistic healing practice that supports the body and mind through the therapeutic use of aromatic EOs. For centuries, the inhalation of volatile compounds from these oils has been a key component of complementary and alternative therapies aimed at promoting mental and physical well-being [74].

One of the most noticeable effects of EOs is their ability to stimulate the senses, particularly through smell. Aromas influence brain activity, and different scents can trigger nerve cells to release various neurotransmitters such as enkephalins, endorphins, noradrenaline, and serotonin. Due to the close connection between the sense of smell and human emotions, EOs have the potential to influence both mental and physical well-being. Scents can evoke emotional responses and alter mood states in individuals [75].

Unsurprisingly, the competitive world of sports has also explored how EOs may support athletes in enhancing their performance. Research suggests that pure EOs have synergistic effects on body and mind energy, facilitating excellence in sports and fitness. Beyond performance, they are also valued in sports medicine for their well-documented antiseptic, anti-inflammatory, pain-relieving, antidepressant, and even expectorant properties [76].

Because of their wide range of physical and psychological benefits, EOs are increasingly being used to tone the body and mind in various ways—supporting muscle preparation, musculoskeletal health, injury prevention, sports medicine, and uplifting sports psychology to achieve peak performance. In this way, using EOs not only aids in preventing and healing sports injuries but also calms and invigorates both mind and body, ultimately enhancing overall physical fitness [77].

Recovery, a top priority for athletes and trainers, is another area where EOs are gaining traction. Professional athletes, in particular, have turned to them for post-exercise recovery, as they can reduce fatigue, restore energy levels, and shorten recovery time. Studies also show that intense physical activity temporarily suppresses immune function; however, certain oils such as lavender and oregano have been found to help maintain immune cell levels, counteracting the usual decline in lymphocyte counts and supporting immune resilience [78].

As highlighted above, aromatherapy has various applications in sports performance [79], and it can generally be divided into two primary categories depending on the method of application: topical aromatherapy and inhalation aromatherapy [80]. The topical aromatherapy involves methods such as massage therapy, medical treatments, bath soak, and cosmetic applications, while the inhalation aromatherapy includes psycho-aromatherapy and olfactory stimulation [80].

EOs commonly used in sports aromatherapy include a variety of plant-based fragrances known for their performance-enhancing and recovery-supporting properties. For example, *Lavandula angustifolia* (lavender) and *Salvia rosmarinus* (rosemary), both representatives of the Lamiaceae family, aid in post-training recovery, help reduce stress hormone levels, and relieve muscle spasms. Lavender oil effects are likely attributed to its active compounds, linalyl acetate, and linalool, which are known for their ability to relieve pain and inflammation, to prevent muscle spasms, and as well as to ease tension [75]. Oils from the Rutaceae family, such as *Citrus limon* (lemon) and *Citrus sinensis* (sweet orange), are associated with the improved performance during repeated high-intensity exercise [64]. Additionally, *Cymbopogon citratus* (lemongrass) from the Poaceae family has been found to enhance athletic performance, to support lung function, to sharpen cognitive focus, and to influence mood positively [64].

- Inhalation aromatherapy:

The lamp diffusion method is one of the simplest and most commonly used techniques for inhalation aromatherapy. As the scent disperses through the air, it can be experienced by anyone in the surrounding area. This method delivers rapid effects, making it especially useful for relieving psychological stress or enhancing mood and emotional states [79].

Inhalation aromatherapy, through olfactory stimulation, primarily functions by activating the olfactory nerve, which extends from the nose to the brain [80]. In addition to this direct olfactory pathway, EOs can also influence brain function via alveolar absorption in the lungs. This process allows volatile EO molecules to enter the bloodstream, cross the blood–brain barrier (BBB), and interact with specific regions of the brain. Lipophilic EO compounds are particularly capable of crossing the BBB and targeting specific areas within the central nervous system, potentially inducing beneficial psychological and physiological effects that help alleviate symptoms of mood disorders [81].

According to some studies, athletes who used peppermint EO before experiencing physical activity achieved notable improvements in lung performance [79,82]. Moreover, inhaling peppermint EO has been shown to enhance grip strength, improve high and long jump performance, boost pulmonary function, relax bronchial smooth muscles, and increase both oxygen intake and mental focus. Peppermint EO is primarily composed of menthol and menthone, along with other compounds such as menthyl acetate, 1,8-cineole, limonene, β-pinene, and β-caryophyllene [83].

- Topical aromatherapy—bath soak

EOs are commonly used in bath soaks to promote relaxation, ease muscle tension, improve circulation, and support skin health. During the bath (full-body or partial immersion), the active compounds in the oils enter the bloodstream through the skin’s sebaceous and sweat glands, as well as through inhalation [84]. The ideal water temperature is around 40 °C, and each session should last between the time gap of 15 and 30 min. To preserve the therapeutic effect, soaps and foaming agents should be avoided. After bathing, the body should be rinsed thoroughly and dried completely [84]. These baths can be enhanced with hydro-massage or underwater massage techniques. Aromatherapeutic baths are used to treat a variety of conditions affecting the skin, nervous system, and cardiovascular system, and they can also help relax or tone muscles after sport [84]. Literature reports suggest that by adding diluted in a carrier oil and incorporated into a warm bath approximately 10–12 drops of EOs such as lavender, eucalyptus, peppermint, marjoram, or ginger, may contribute to the alleviation of muscle soreness, reduction in inflammation, and promotion of post-exercise recovery. Within the context of sports medicine and, in particular, in winter sports, specific EOs are described for targeted effects: rosemary oil for reducing fatigue, black pepper oil for thermogenic and warming properties, and lemon or lemongrass oil for enhancing energy and athletic performance [85].

- Topical aromatherapy—massage therapy:

When applied topically using massage therapy, EOs can produce analgesic effects and promote a sense of comfort, primarily due to the rapid release of endorphins and various pain-modulating substances [80]. The term “massage”, likely originating from the Greek word “*massein*”, meaning “to knead”, now refers to a broad range of therapeutic and relaxation techniques involving manual manipulation of the body. These techniques are often combined with the inclusion of natural elements such as herbs, EOs, water, salts, or mud. Aromatherapy massage, also known as “aroma massage”, or “fragrant massage”, is a therapeutic technique that takes EOs to deliver active compounds into the body. This practice has been used for many years and typically consists of diluting EOs in a carrier oil such as sweet almond, grapeseed, calendula, jojoba, olive, sesame, sunflower wheat germ, or peach oil for direct application to the skin [86] in concentrations ranging from 1.5% to 3.0%. The carriers utilized in aromatherapy massage should effectively be able to dissolve EOs, to be fresh, and preferably to have minimal or no scent [87]. When applied to the face, where the skin is more delicate, dilution should be reduced to 0.2–1.5%, to minimize the risk of adverse reactions [88]. As noted in Wani et al.’s 2021 study, massage therapy is often described as a form of “healing touch” [89] because aromatherapy massage combines the therapeutic power of touch with the healing properties of essential and carrier oils. In professional practice and in sport, a typical session uses 15–30 drops of EOs blended with 50 mL of carrier oil. This mixture can be prepared for a full-body massage or for localized applications. The choice of EOs depends on the therapeutic effects desired [90,91].

In addition to training, massage is often included in the preparation process of the athletes for competition. Pre-event massage can help lower anxiety and condition the muscles, and when paired with essential oils, it may further reduce injury risk and boost psychological readiness. Post-exercise massage incorporating EOs has been reported to alleviate delayed-onset muscle soreness and facilitate recovery, potentially through enhanced peripheral blood circulation. Targeted massage techniques, when combined with EOs possessing analgesic (lavender oil) and anti-inflammatory (eucalyptos and peppermint oil) properties, may further contribute to reducing chronic musculoskeletal pain—particularly in the back, shoulders, and lower limbs—thereby improving mobility and relieving muscle tension [92].

#### 2.2.2. Topical Application of EOs as Medical Treatment

When applied to the skin, EOs are absorbed, and their active compounds are utilized by the body for targeted therapeutic effects. Proof about that statement may be found in the following section. For example, using ginger EO has been shown to relieve arthritis pain and enhance joint flexibility [79]. This is due to EO constituents’ interaction with various transient receptor potential (TRP) channels, which play key roles in the perception of pain, heat, and cold. Therefore, they are very helpful in sports medical treatment. Their topical application may cause musculoskeletal pain and inflammation to be reduced, blood circulation to be enhanced, and cooling and local anesthetic effects to be provided. Additionally, they promote muscle relaxation [93].

EOs topical formulations are available in various forms, including liquid systems and semi-solid forms. EO ointments are semi-solid, anhydrous preparations composed of immiscible bases and EOs, primarily intended for topical application to the skin [94]. For example, Ping On Ointment (manufactured by Ping On Ointment Company Limited, Hong Kong) is a registered topical medicinal balm [95]. Its primary ingredients include peppermint oil (18%), menthol (20%), natural camphor (6%), birch oil (6%), sandalwood oil (1%), eucalyptus oil (4%), beeswax (8%), and aromatic oil (3%). The formulation is free of antibiotics, steroids, cortisone, and preservatives. Registered in Hong Kong since 1965, it has been widely used as a soothing balm for relieving muscle aches, strains, and sprains [95]. Research on the anti-inflammatory and pain-relieving properties of eucalyptus EOs has demonstrated that they exhibit both neutrophil-dependent and independent anti-inflammatory actions, along with analgesic effects that act on both the central and peripheral nervous systems [95]. The study of Larry et al. (2009) showed that the topical use of this ointment may effectively reduce the severity of muscular pain associated with Temporomandibular Disorder (TMD), suggesting it could serve as a promising alternative treatment for TMD-related muscle discomfort [95].

EO creams are semi-solid dosage forms containing both hydrophilic and hydrophobic bases in which EOs are incorporated. Compared to ointments, they typically have a shorter shelf life due to their higher water content [94].

Ou et al. (2014) developed a cream containing a 3% blend of four EOs—marjoram, black pepper, lavender, and peppermint—and evaluated its effectiveness in relieving neck pain. Sixty individuals with a history of neck discomfort and a Neck Disability Index (NDI) score above 10% were recruited and randomly assigned to either an experimental or control group. Over a four-week period, all participants applied 2 g of the EOs cream daily to the affected area following a shower or bath. Results indicated that the group using the EOs cream experienced greater relief from neck pain compared to the control group, which used a cream without EOs [96].

EOs balms are similar to ointments in consistency and formulation but are specifically designed for the relief of pain. They are prepared by blending EOs with a suitable ointment base [94].

Aromatic waters are aqueous solutions infused with EOs, typically prepared by blending distilled water with EO and talcum powder or by micellar solubilization with Tween [94]. Common examples include rose water, lavender water, cardamom water, peppermint water, and camphor water [97].

EOs liniments are liquid formulations containing EOs, intended for external use and applied with friction (rubbing) to relieve muscular or ligament pain and discomfort [94].

EO emulsions are heterogeneous dispersion systems composed of two immiscible liquids, typically oil and water, where one liquid is dispersed within the other in the form of fine droplets. EO emulsions generally consist of an oil phase containing EOs, an aqueous phase, surfactants to stabilize the emulsion, and additional functional additives as needed. Emulsions are generally classified into two types: oil-in-water (*o*/*w*) and water-in-oil (*w*/*o*) systems. They are among the most commonly used delivery systems for EOs, as they help reduce oxidation, mask strong odors, and improve water solubility [98].

At room temperature, EOs exist in liquid form. As a result, the simplest method of encapsulation involves emulsifying or dispersing them in an aqueous solution. However, this liquid formulation can be challenging to handle. A practical solution is to convert it into a dry form through micro- or nanoencapsulation, where the oil droplets are trapped within a carrier material. Encapsulation techniques for EOs are generally categorized into three main groups: chemical methods (such as molecular inclusion and interfacial polymerization), physicochemical methods (including coacervation and liposome encapsulation), and physical methods (like spray drying, spray chilling or cooling, co-crystallization, extrusion, and fluidized bed coating) [99].

In recent decades, considerable attention has been directed toward the development of novel drug delivery systems for the topical administration of EOs by using the above-mentioned techniques. These advanced systems include polymeric nanoparticles, nanoemulsions, microspheres, phytosomes, nanocapsules, and others [100]. Additionally, carriers such as ethosomes and transferosomes have been employed to encapsulate EO-derived bioactive compounds [101]. Encapsulating EOs has emerged as an effective strategy to overcome the limitations that hinder their broader pharmaceutical use. Compared to conventional formulations, these innovative systems offer numerous advantages, including enhanced solubility, bioavailability, and pharmacological activity, as well as reduced toxicity, improved stability, targeted tissue distribution, sustained release, and protection from physical and chemical degradation. Notably, recent advances have explored the co-encapsulation of EOs with conventional synthetic drugs, aiming to boost therapeutic effectiveness, enhance biocompatibility, and reduce drug resistance. As highlighted, the formulation’s qualitative and quantitative composition, along with the chosen preparation method, are critical factors in developing an optimal product tailored for applications in sports and physical performance [100].

Lora et al. (2022) developed a performance-enhancing sports T-shirt infused with eucalyptus EO [102]. The garment is designed to offer a refreshing sensation and help clear the airways, while also inhibiting the growth of odor-causing bacteria due to the antibacterial properties of eucalyptus oil. The product is aimed primarily at athletes and outdoor enthusiasts, such as hikers. The manufacturing process involves three main stages: extraction of the EO, microencapsulation, and application to the fabric. Eucalyptus oil was extracted using supercritical carbon dioxide, chosen for its efficiency and purity. Interfacial polymerization was recognized as the most efficient technique for microencapsulating the oil. Finally, the microcapsules were attached to the fabric using the exhaustion method [102].

### 2.3. Clinical Studies Investigating the Relationship Between Athletic Recovery/Performance and Essential Oils

Although many studies investigated the biological activity of EOs and the application of EOs in the treatment of many medical conditions, including depression, anxiety and others, we have identified only 12 studies that investigated the effects and benefits of EOs in sports medicine (Table 2). Only selected examples have been analyzed in detail. The main focus of these studies was the support of sports performance, recovery and injury prevention.

The investigations regarding the therapeutic benefits of EOs in sports medicine are quite limited, primarily focusing on peppermint and lavender EO. While several trials suggest peppermint EO may improve endurance or motor performance [33,73,110], the overall evidence is inconsistent. The positive endurance outcome in [33] was observed in a small sample (n = 14) of recreational male runners. In contrast, the study by [103] found no significant performance benefit despite similar dosing, possibly due to differences in participant fitness level, intervention duration, or performance endpoints.

For lavender EO, two studies [111,112] reported reduced fatigue and improved sleep quality. However, both studies relied on self-reported outcomes without objective physiological markers (e.g., actigraphy, cortisol levels). Moreover, neither peppermint nor lavender studies applied long-term follow-up to determine whether benefits persist beyond the acute phase.

Across the 12 identified trials, common methodological weaknesses include small sample sizes (most n < 40), lack of standardized EO dosing, heterogeneous outcome measures, and inadequate blinding for aromatic interventions. These factors hinder meta-analytic synthesis and reduce the strength of evidence. Future trials should employ standardized EO chemotypes, objective performance metrics, and larger, more diverse athlete cohorts.

### 2.4. Application of Essential Oils—Precautions and Limitations

It is crucial to recognize that “natural” does not necessarily equate to “safe” [113]. EOs represent complex mixtures, containing numerous compounds. Some of them are associated with strong sensitizing effects (citral, α-terpinen, etc.). For instance, the monoterpene citral is a key fragrance compound found in the EOs of many plant species. According to the EU Cosmetic Products Regulation (EC) (No. 1223/2009), citral must be listed on cosmetic product labels if its concentration exceeds 0.001% in leave-on and 0.01% in rinse-off products. Similar labeling rules apply to other allergens like citronellol, limonene, and α-terpinene. Limonene and α-terpinene must also meet peroxide value limits of less than 20 mmol/L. These compounds are classified as potential allergens. Their ability to cause skin sensitization is linked to autoxidation, during which they form reactive compounds. For example, pure citronellol is typically safe, but when exposed to air, it oxidizes into hydroperoxides, which can trigger allergic reactions. These hydroperoxides are thought to interact with skin proteins via radical-mediated mechanisms. However, the exact pathways of sensitization are not yet fully understood [114].

Since the topical application of EOs (especially in massage after exercise or injury) is part of aromatherapy, some disadvantages associated with this application include skin irritation, skin sensitization, photosensitization, skin inflammation (such as eczema and dermatitis—caused by tea tree oil, citrus oils, mint oils, and tree-based oils), and allergic reactions (such as urticaria—tea tree oil, ylang-ylang, sandalwood, lemongrass, jasmine absolute, clove, lavender, and peppermint). EOs should not be applied to severely injured skin (for example, after trauma or abrasion), so as not to increase the area of inflammation. They are usually diluted in carrier oils or in oil creams before application to avoid skin irritation or sensitization [100,115].

The therapeutic effect sometimes occurs more slowly than with a similar form of medicine. Due to the lack of regulation by the Food and Drug Administration (FDA), the quality, purity, and efficacy of EOs can vary significantly depending on factors such as plant source, extraction technique, and storage conditions. The physiological effects of essential oils may vary from person to person, influenced by variables including dosage and the specific combination of oils used. Drug interactions: Some EOs may interact with medications or herbal products, potentially enhancing or decreasing their therapeutic effects. Adverse reactions: Inappropriate or excessive use of EOs may result in adverse reactions such as nausea, headache, fatigue, or photosensitivity. For example, citrus-based EOs may cause skin irritation or burns upon exposure to sunlight or ultraviolet (UV) radiation due to their photosensitizing compounds (furanocoumarins), especially in athletes who train outdoors, where there is increased skin absorption and sweating [100,115].

Some EOs can exhibit toxicity at high concentrations. Their potential toxicological profile may differ significantly from that of the whole plant from which they are derived, not only due to their concentrated nature but also because of their high lipophilicity, which allows them to readily cross biological membranes [116]. The potential toxic effects of certain EOs and their components have been examined in laboratory animals, primarily rodents. Acute toxicity is commonly assessed using the LD50 test (median lethal dose) in rats. Results have shown that most EOs have an LD50 ranging from 1 to 20 g/kg, suggesting relatively low toxicity. In humans, EOs such as lemon oil exhibit an LD50 above 5 g/kg. For a 70 kg adult, this would equate to a lethal dose of approximately 350 g—an amount unlikely to be reached under normal conditions [117,118]. To minimize the risk of acute poisoning, EOs should be stored in child-resistant containers with droppers and kept separate from oral medications to prevent acute toxicity [117].

To minimize these risks, it is essential to use EOs under the guidance of a qualified professional (such as trainers, masseurs, rehabilitators, sports doctors, pharmacists, paramedics), adhere to recommended dosages and duration, and avoid direct sunlight or heat exposure following topical application [115].

Moreover, even EOs generally regarded as safe may cause adverse effects in certain individuals, particularly in cases of prior sensitization. Given their considerable biomedical potential, it is crucial—especially in pharmaceutical applications—to possess a comprehensive understanding of the specific EO’s properties, including its pharmacodynamics, pharmacokinetics, and potential toxicities, prior to therapeutic use in sports and physical activities [116].

Research showed that most EOs are quickly absorbed through the skin, orally, or via inhalation, and they can cross the BBB to interact with receptors in the central nervous system, thereby influencing important biological functions. However, many components of EOs are rapidly metabolized, resulting in a short half-life and, in some cases, low bioavailability. Bioavailability refers to the portion of a substance that successfully reaches systemic circulation. When a compound undergoes extensive metabolism, its absorption is limited, meaning only a small fraction of the administered dose enters the bloodstream. Fortunately, advancements in drug delivery technologies are now helping to overcome these bioavailability challenges by utilizing innovative delivery systems [119].

The degradation of EOs is influenced by a combination of chemical and environmental factors, which affect both the oxidation and the progression of degradation reactions. External conditions such as temperature, light exposure, and access to atmospheric oxygen must be carefully considered. In addition, the stability of EOs is also determined by their chemical composition, the structural characteristics of their constituents, and the presence of impurities [120]. Ultraviolet and visible light are known to accelerate autoxidation by initiating hydrogen abstraction, which leads to the formation of alkyl radicals [121]. Exposure to light enhances autoxidation by facilitating the formation of alkyl radicals and catalyzes intramolecular isomerization processes, including trans–cis conversions in monoterpenes, thereby accelerating their degradation. Similarly, heat intensifies chemical reactions, leading to the formation of primary autoxidation products such as hydroperoxides. As the temperature continues to rise, these hydroperoxides break down further, producing final oxidation products [120]. Since oxygen in the headspace gradually diffuses into the sample during storage, EOs should be stored in filled containers. Alternatively, treating the container with an inert gas to displace residual air can help prevent oxidative degradation [122].

Encapsulation of bioactive compounds presents a practical and effective strategy to control drug release, enhance the physical stability of active ingredients, shield them from environmental exposure, reduce volatility, improve bioactivity, lower toxicity, and enhance patient compliance and usability. A significant portion of recent research on EOs encapsulation focuses on the use of micro- and nanoscale capsules. These systems serve to protect active compounds from external factors such as oxygen, light, moisture, and pH, while also minimizing volatility and enabling the conversion of EOs into more manageable powder forms. Encapsulation at the nanoscale, in particular, not only addresses these challenges but also offers the added benefit of improving cellular uptake due to their subcellular size, thereby potentially enhancing bioefficacy [123]. Encapsulation of EOs in novel drug delivery systems has been shown to mitigate such side effects by improving stability and controlled release. Research showed that lipid-based drug delivery systems—including micro/nanoemulsions, liposomes, solid lipid nanoparticles, and nanostructured lipid carriers—enhance the stability, efficacy, and bioavailability of EOs compared to their unformulated forms [100].

The chemical profiles of EOs from the same species can vary significantly based on factors such as the plant geographic origin, the specific part of the plant used, its growth stage, and surrounding ecological and climatic conditions, extraction techniques, and drying methods. At present, multiple standards and guidelines address the quality of EOs, including ISO 4730:2017, the French Standard T75-358, the European Pharmacopeia, the British Pharmacopeia, and others. Despite this, recent research indicates that EOs marketed as cosmetic products often do not fully comply with the quality criteria outlined in these official standards [124].

## 3. Materials and Methods

The initial phase of the screening process involved identifying eligible studies through an extensive search of multiple databases, including PubMed, Scopus, Web of Science, and Google Scholar. A comprehensive set of keywords was employed to maximize the retrieval of relevant literature. The keywords included the following: essential oils, recovery, sport, athletic performance, muscle recovery, mental health, aromatherapy, aromatic plants, sport medicine, peppermint essential oil, rosemary essential oil, eucalyptus essential oil, lavender essential oil, orange oil, lemon oil, arnica oil.

In the final phase, studies were selected based on predefined inclusion and exclusion criteria. Exclusion criteria encompassed non-peer-reviewed sources such as webinars, blogs, and articles lacking sufficient data, studies involving animals, studies involving adults suffering from chronic diseases, studies involving children, and studies involving pregnant or postpartum women. Inclusion criteria required studies to involve healthy adult participants and to examine the relationship between EOs and recovery, or athletic performance. We have identified 12 clinical studies investigation the relationship between EOs and sports medicine. We acknowledge that the majority of available studies on aromatherapy are limited by small sample sizes, lack of rigorous control groups, and reliance on subjective outcomes.

## 4. Conclusions

Although EOs have a long history of traditional use, the clinical evidence supporting their application in sports performance and recovery remains limited and heterogeneous. Among the available data, peppermint and ginger EO show relatively stronger evidence, with findings indicating potential benefits for endurance, respiratory function, and attenuation of exercise-induced inflammation and soreness. In contrast, EOs such as lavender, rosemary, eucalyptus, and citrus species are supported primarily by preliminary studies, often relying on subjective outcomes or indirect measures such as mood, sleep quality, or perceived fatigue. Key limitations of the current studies include small sample sizes, short intervention periods, heterogeneity in EOs chemotypes and dosing regimens, insufficient use of objective physiological and performance endpoints. Moreover, the chemical composition of the studied EOs was not analyzed. Future research should prioritize multi-center randomized controlled trials with standardized EOs preparations, rigorous blinding, and validated biomarkers of inflammation, muscle recovery, and athletic performance. Comparative studies against established recovery strategies, as well as investigations into delivery systems and long-term outcomes, are essential to determine whether EOs can be credibly integrated into evidence-based sports medicine.

## Figures and Tables

**Table 1:** Important aromatic plants for sport medicine.

Essential Oil	Aromatic Plant (Botanical Name)	Main Volatile Compounds Found in the EOs	Key Uses	Benefits	Applications	Refs.
**Arnica**	*Arnica montana* L.	Achenes: Cumene, Thymol Methyl Ether, 2,5-Dimethoxy-*p*-cymene, 2,6-Diisopropylanisole. Roots and Rhizomes: 2,5-Dimethoxy-*p*-cymene, 2,6-Diisopropylanisole, Thymol methyl ether, *p*-methoxyheptanophenone.	Bruise and injury recovery, muscle soreness.	Reduces inflammation and speeds up healing of soft-tissue injuries.	External application only.	[15,16]
**Chamomile**	*Matricaria chamomilla* L.	Flowers: (*E*)-β-Farnesene, (*E*,*E*)-α-Farnesene, α-Bisabolol oxide A, Chamazulene, α-Bisabolol oxide B, Germacrene D, (*Z*)-Spiroether.	Muscle pain relief.	Reduces pain intensity after orthopedic surgeries. Reduces inflammation and accelerates the wound healing process.	Topical application.	[17,18,19,20]
**Cinnamon**	*Cinnamomum verum*	Leaf and flower: (*E*)-Cinnamaldehyde, Eugenol, Linalool, (*E*)-Cinnamyl acetate. Bark:(*E*)-Cinnamaldehyde, Eugenol, Linalool, α-Pinene.	Anti-inflammatory, muscle recovery, muscle soreness, increases endurance, energy, and circulation booster.	Prevents/reduces inflammation. Reduces muscle discomfort and oxidative damage after training. Improves circulation and brain activity.	External application only.	[21,22,23,24,25,26]
**Eucalyptus**	*Eucalyptus globulus*	Leaves: 1,8-Cineole, *p*-Cymene, α-Pinene, Limonene.	Respiratory aid, anti-inflammatory, pain relief, wound healing, treatment for muscle strains and sprains.	Positively influences respiratory diseases. Reduces inflammation and pain. Accelerates the wound healing process. Relieves joint and muscle discomfort.	Inhalation, balms, creams.	[27,28,29]
**Ginger**	*Zingiber officinale* Rosc.	Rhizomes: Zingiberene, Citral, β-Bisabolene, Geranial, Camphene.	Muscle pain relief, anti-inflammatory activity, muscle recovery, muscle soreness.	Anti-inflammatory activity. Analgesic and antioxidant activity.	Topical use and aromatherapy.	[30]
**Lavender**	*Lavandula angustifolia* Mill.	Aerial parts: Linalyl acetate, Linalool, Lavandulol acetate.	Muscle relaxation, stress reduction. Improves the quality of sleep.	Anti-inflammatory activity. Calming effects for nervous system. Reduces cortisol levels.	Aromatherapy, massage, bath soaks.	[31]
**Peppermint**	*Mentha piperita* L.	Aerial parts: Menthol, Menthyl acetate, Menthofuran, 1,8-Cineole.	Muscle pain relief, energy boost, respiratory support.	Improves circulation. Cooling sore muscles. Improves focus.	Topical creams, massage, inhalation before workouts.	[32,33,34,35]
**Rosemary**	*Salvia rosmarinus* Spenn.	Herba: α-Pinene, Eucalyptol, 3-Camphor, endo-Borneol.	Mental clarity, muscle soreness, and muscle spasms, circulation booster.	Reduces stress hormone levels. Enhances fatigue. Alleviates muscle spasms.	Aromatherapy, massage, bath soaks.	[1,36,37,38]

**Table 2:** Clinical studies regarding sport medicine and essential oils.

EO	Population	Study Design	Intervention	Control	Outcomes	Main result	Ref.
Peppermint (PEO)	14 male recreational runners (average age: 37 ± 2.0).	Randomized, double-blind, cross-over, controlled clinical trial.	Each participant completed 2 endurance runs to exhaustion at 70% of their VO_2_ max. Before each run, they consumed 500 mL of water containing either 0.05 mL of PEO or a placebo, along with an additional 400 mL of the same drink during the early phase of exercise.	Measured thermal sensation (TS), thermal comfort (TC), body temperature (BT), sweat rate (SR), subjective perception of effort (SPE), and urine density and volume.	PEO extends the duration until exhaustion for recreational runners, while not impacting body temperature, thermal sensation, core temperature, or hydration levels.	Extended endurance time of recreational runners.	[33]
Peppermint (PEO)	7 healthy active participants (average age: 24.57 ± 3.95 years).	Randomized, single-blind, cross-over design.	Conducted research with two groups: Group 1—received PEO for 10 days, washout period of 7 days, followed by 10 days of control supplementation; Group 2—control supplementation first for 10 days, and PEO after for 10 days.	Effect on aerobic performance.	No notable differences were found in expired gas variables (peak oxygen uptake) and performance metrics (time to exhaustion) between the PEO group and the control group. The cardiopulmonary resting measurements remained consistent across visits.	No significant differences were observed.	[103]
Peppermint, Rosemary, Eucalyptus.	106 healthy participants (average age: 21.2 ± 2.56 years).	Randomized, controlled trial.	Received nebulized PEO, eucalyptus, or rosemary EO for 15 min or no treatment (control).	Effect of the EOs on spirometry measures.	No significant impact was observed on the measured peak expiratory flow, forced vital capacity, the ratio of the volume of air forcibly blown out in the first to second forced vital capacity. The anticipation of the participants did not influence their actual performance, but they anticipated subjective changes.	No effect on spirometry measurements in healthy individuals was indicated.	[104]
Grapefruit	Study 1: 13 healthy male participants (average age: 21 ± 2.1 years). Study 2: another 9 healthy male participants (average age: 21 ± 2.2 years). Study 3: another nine healthy male participant (average age: 23 ± 2.8 years).	Randomized, controlled trial	Study 1: Exposure to aroma stimulation for 10 min. Study 2: Exposure to aroma stimulation for 10 min. Study 3: Exposure to air containing no fragrance (control trial) or to air containing aroma (fragrance trial) for 10 min.	Study 1: Measured sympathetic muscle nerve activity (MSNA), blood pressure (BP), heart rate (HR), respiratory variables, and subjective emotion. Study 2: Measured BP, HR, respiratory variables, and subjective emotion. Study 3: Determined plasma adrenalin (A), noradrenaline (NA), adrenocorticotropic hormone (ACTH), and cortisol concentrations.	Study 1: No significant change in burst incidence and frequency, or total MSNA; increased diastolic BP (DBP); unchanged HR and respiratory variables. The subjective emotion ratings were predominant in the pleasant and relaxing parts of the scale (Study 1 and Study 2). Study 2: Significantly increased DBP; unchanged HR and respiratory variables; unchanged NA levels; significantly decreased cortisol levels. Study 3: Unchanged A and NA levels; decreased cortisol and ACTH levels in the fragrance trial.	Rosen DBP, decreased cortisol levels.	[105]
*Coleus forskholii*, Silybin, *Eucommia ulmoides* leaf, *Paullinia cupana* seed, caffeine, and black pepper.	20 healthy participants (average age: 26.3 ± 6.3 years).	Randomized, double-blind, placebo-controlled trial.	Conducted research with 2 sets of topical lotions (fat loss lotion and a placebo lotion) for each participant, for 8 weeks.	Alterations in subcutaneous fat thickness; recorded body weight and height; overall body fat percentage and leg fat percentage.	No significant difference was observed between the treatment group and placebo group in terms of subcutaneous fat thickness on the anterior thigh, nor in fat percentage in the leg. A minor difference in the lateral thigh of the treated leg was indicated.	No significant differences observed.	[106]
*Origanum dubium* (DUB), *Origanum vulgare* subsp. *hirtum* (HIR), and *Lavandula angustifolia.*	34 trained athletes	Randomized, controlled trial.	Conducted research with 3 experimental groups (DUB, HIR, and lavender) and a control group for 14 days.	Lipid profiles and liver biomarkers before and after intervention.	A significant difference in high-density lipoprotein cholesterol (HDL-C) levels among the groups was observed. Both DUB and HIR exhibited higher HDL-C levels compared to the control group. An interaction was indicated between time and the groups regarding HDL-C. An increase in HDL-C levels was noted in both DUB and HIR, along with a reduction in low-density lipoprotein cholesterol for DUB. No variations in total cholesterol, triglycerides, or any liver biomarkers were found.	Increase in DUB and HIR for high-density lipoprotein cholesterol, decrease in DUB for low-density lipoprotein cholesterol.	[107]
Olibanum, Chuanxiong.	116 participants (average age: 22.05 ± 2.86 years- control group; 21.90 ± 2.30 years- test group).	Randomized, controlled clinical trial.	Conducted conventional ultrasound therapy (control group), and conventional ultrasound therapy and EOs (test group) for 3 days.	Analgesic effect on sports-induced knee synovitis.	The pain levels in both male and female participants diminished in both groups; the Lysholm scores showed improvement in each group; there was a reduction in both the range of motion and the knee circumference following the treatment. No adverse effects or complications were noted during or after the course of treatment.	The combined therapy had a superior analgesic effect to conventional ultrasound therapy.	[108]
Rosemary, Lavender, Eucalyptus.	33 participants (average age: 37.7 ± 10.90 years).	Randomized, double-blind, controlled clinical trial.	Inhalation of the EOs separately, using a silicone face mask, for 3 min each.	Effects on alertness; measured blood pressure (BP), heart rate (HR) and heart rate variability (HRV).	The alertness was affected, which influences the blood pressure, heart rate, and heart rate variability, but it was not correlated with real alterations in the corresponding vegetative states.	Affected alertness.	[109]
Peppermint (PEO)	11 soccer players (average age: 17.5 ± 0.32 years).	Randomized, double-blind, cross-over, placebo-controlled study.	Conducted research with a study group (inhalation of PEO) and placebo group, including 3 visits of the participants.	Impact of PEO on motor performance.	Significant main effects were observed for the time in jumping height in the countermovement jump. There was also a small negative effect size under both conditions for jumping height (decrease); a small positive effect size (increase) in yielding impulse—PEO group; a small negative effect size (decrease) in yielding impulse—placebo group. No main effects or interaction effects were detected in the squat jump variables.	PEO affects the time in jumping height in the countermovement jump.	[73]
Peppermint (PEO)	48 healthy male participants (age: 20–30 years).	Randomized, single-blind, controlled trial.	Conducted research with a study group (inhalation or uptake—50 μL of PEO), and a control group (no treatment) for 10 days.	Effects on the autonomic, cardiovascular, respiratory, and metabolic systems; impact on athletic performance.	No significant differences were observed in the anthropometric parameters. Increased sympathetic activity and sympathovagal balance in the uptake group, and no difference in the inhalation and control groups. No significant difference in exercise time, VO_2_ (oxygen consumption), VCO_2_ (carbon dioxide production), V_E_ (ventilation per minute), Vt (tidal volume), RR (respiratory rate), V_E_/VO_2_, and V_E_/VCO_2_. However, VCO_2_ max, V_E_ max, RR max, and V_E_/VCO_2_ significantly increased in the uptake tested group.	The increased sympathetic activity at rest could be connected to respiratory control during high-intensity workouts.	[110]
Lavender (LEO)	60 student-athletes (age: 18–25 years).	Randomized controlled trial.	Conducted research with a study group (sauna infused with LEO), and a control group for 15 min post-exercise.	Impact of the LEO on fatigue levels and performance.	Reduced average lactate levels were observed in the study group, leading to decreased fatigue and pain conditions. Notable variations in pulse values during the first minute, alongside saturation during the fifth minute. Reduced blood sugar levels in both groups compared to the initial value.	Reduced fatigue and relieved painful conditions.	[111]
Lavender (LEO)	42 trained athletes (age: ≥18 years).	Randomized parallel-group controlled trial.	Conducted study with 4 groups: control group (CG), sleep hygiene education group (SHEG), lavender EO group (LEOG) inhalation, and a combined SHE and LEO group (CSLG), for 7 days after nighttime strength training.	Impact of the LEO on sleep quality and emotional states.	Sleep latency was reduced in both SHEG and CSLG compared to CG. Subjective sleep scores improved in SHEG, LEOG, and CSLG compared to CG after the intervention. Advancements were observed in the Hooper index for SHEG and CSLG. A significant reduction in subjective fatigue score was noted for CSLG.	Reduced fatigue, enhanced subjective sleep quality, and overall wellness.	[112]

## Data Availability

Data is contained within the article.
